# Finding Harmony between Science and Art in Pediatric Cardiology: Acknowledging When Being “Objective” May Not Truly Be Objective

**DOI:** 10.3390/children3040037

**Published:** 2016-11-23

**Authors:** Rohit S. Loomba

**Affiliations:** Children’s Hospital of Wisconsin/Medical College of Wisconsin, 9000 Wisconsin Avenue, Milwaukee, WI 53226, USA; loomba.rohit@gmail.com; Tel.: +1-630-881-8342

As pediatric cardiologists, we greatly value objective decision-making and logic. In fact, it is the physics-driven nature of pediatric cardiology that attracts many professionals to the field. This desire for data-driven decisions becomes even greater based on the degree of severity that patients with congenital malformations of the heart may suffer. This phenomenon manifests itself in both the clinical and academic realms of the field and can often impact the perception of delivered care or conducted research. Because of this, we must explore what our framework of objectivity is, why we strive for increasing the science behind our decisions while minimizing the art in our decisions, and whether or not this maximization of science with minimization of art is truly even helpful.

In clinical pediatric cardiology, objectivity is most often synonymous with quantitative measures: laboratory data, oximetry data, hemodynamic data, and vital signs. In research, objectivity is similarly focused around quantitative assessment. Qualitative assessments such as various assessments made by echocardiography, for instance, are often dismissed on the basis of being subjective and replaced by quantitative assessments, which are felt to be more objective. However, the question remains if quantitative measures are always truly better than qualitative measures? For instance, is measuring the vena contracta of a regurgitant jet by echocardiography truly more accurate than an experienced echocardiographer grading the degree of insufficiency? Can we be entirely certain the narrowest portion is indeed in the plane of imaging that they are using? Can this even be achieved without obtaining a true three-dimensional reconstruction that can be interrogated in the infinite number of planes that are present? Can we be entirely certain that other fluid jets aren’t impacting the so-called vena contracta? Another example lies in the concept of assessing left ventricular function. Those who have performed echocardiograms are well aware that shortening the fraction and ejection fraction can change significantly with subtle changes in the actual tracing of the ventricular boundary on the echocardiographic imaging. Tracings can appear to reasonably and equally approximate these boundaries, but can produce values that fall on either side of the cutpoint that is defined as normal. Not to mention that these cutpoints are human-defined and prone to error in our own understanding and are also the product of human-developed statistics. So then why do we insist that the decision to shorten fraction and ejection fraction is more reasonable than qualitative assessments of function by an experienced echocardiographer?

Quantitative measures, even those that are accurately reproducible, are dependent upon human interpretation, which, itself depends upon the health-care providers’ understanding of statistics, and is thus limited by their level of statistical expertise or understanding. Additionally, normal values are determined by their value’s position inside a specific range that contains a majority of data points. The number of data points, be it 92.5%, 95%, or 97.5%, is often arbitrarily selected by researchers who have decided that a certain percentage of values are abnormal based on just the fact that they fall in a certain tail area of a distribution. This can present a particular selection bias since, when it comes to interpreting research manuscripts, many fail to understand what *p*-values actually are. The *p*-value represents the percent likelihood that the null hypothesis may be rejected, because the noted association is actually the result of random chance. Technically speaking, there is no objective way that statisticians have defined this cutoff. A particular cutoff level was simply decided as acceptable, and consequently affects what research results are considered statistically significant. A cutoff of 0.05, which is often used as the threshold separating statistical significance from statistical insignificance, has become universally accepted by healthcare providers and researchers as a generally acceptable value, even when the data may warrant a different value. In fact, it is sometimes appropriate to change the level of significance for certain studies, and researchers will do this, using values of 0.025 or 0.1. Often, however, these manuscripts are dismissed as being statistically unsound by those who do not have a true understanding of the necessity for the change in *p*-value. Thus, it becomes evident that even the statistical system that is chosen or accepted, and that we feel helps us objectively represent findings, can be based on arbitrary human assumptions. Thus, it could be argued that an objective interpretation is actually based on a subjective system.

The point of this discussion is not to say that we should give up using a statistical analysis or the scientific method, but rather that we must be more aware that what we do in pediatric cardiology must represent a blend of science and art, namely that we must support the validity of both qualitative and quantitative assessments. Are cardiothoracic surgeons not the highest degree of artists? No echocardiographic parameters can tell them exactly how to separate the common atrioventricular junction in the setting of an atrioventricular septal defect to ensure there is neither stenosis nor regurgitation, yet they must be able to do this in the operating room using subjective assessments of experience and instinct. Similarly, we must be able to embrace this and understand that even what we think are the most objective of measures can be prone to human subjectivity.

Having an entirely science-based practice isn’t practical. We cannot ignore human instinct and emotion in any of our endeavors. Particularly in a field such as pediatric cardiology, it becomes of utmost importance that we understand that science and art aren’t separable entities. They are both in finest form when they are in balance with one another. Our goal should focus on achieving the proper balance of science and art, such that clinical outcomes are optimized, not eliminating art entirely.

